# Vitamin K epoxide reductase and its paralogous enzyme have different structures and functions

**DOI:** 10.1038/s41598-017-18008-3

**Published:** 2017-12-15

**Authors:** Balaji Chandra Sekhar Sinhadri, Da-Yun Jin, Darrel W. Stafford, Jian-Ke Tie

**Affiliations:** Department of Biology, University of North Carolina at Chapel Hill, Chapel Hill, North Carolina 27599 United States of America

## Abstract

Vitamin K epoxide reductase (VKOR) is an essential enzyme for vitamin K-dependent carboxylation, while the physiological function of its paralogous enzyme VKOR-like (VKORL) is yet unknown. Although these two enzymes share approximately 50% protein sequence homology, the membrane topology of VKOR is still in debate. Here, we explored the differences in the membrane topology and disulfide-linked oligomerization of these two enzymes. Results from mutating the critical amino acid residues in the disputed transmembrane (TM) regions revealed that the second TM domain in the proposed 4-TM model of VKOR does not function as an authentic TM helix; supporting VKOR is a 3-TM protein, which is different from VKORL. Additionally, altering the loop sequence between the two conserved cysteine residues of VKORL affects its activity, supporting the notion that the conserved loop cysteines of VKORL are involved in its active site regeneration. However, a similar mutation in VKOR does not affect its enzymatic activity. Finally, our results show that although both VKOR and VKORL form disulfide-linked oligomers, the cysteine residues involved in the oligomerization appear to be different. Overall, the structural and functional differences between VKOR and VKORL shown here indicate that VKORL might have a different physiological function other than recycling vitamin K.

## Introduction

Vitamin K is a family of 2-methyl-1,4-naphthoquinone derivatives that includes the naturally occurring menaquinones and phylloquinone. In mammals, the primary function of vitamin K is to act as a cofactor for the enzyme gamma-glutamyl carboxylase (GGCX)^1^. About 70% of vitamin K absorbed from the diet is lost to excretion. Therefore, the body’s stores of vitamin K are recycled in a process known as the vitamin K cycle^1,2^. In the vitamin K cycle, an endoplasmic reticulum (ER) membrane protein called vitamin K epoxide reductase (VKOR) reduces vitamin K 2,3-epoxide (KO) to vitamin K (K); this vitamin K is then further reduced to vitamin K hydroquinone (KH_2_) by a yet-unknown enzyme^3,4^. VKOR is the target of the popular vitamin K antagonist (VKA) drug warfarin, which is widely used to treat thrombosis^5^. Warfarin inhibits the activity of VKOR, thereby preventing the conversion of KO to KH_2_. KH_2_ is a necessary cofactor for GGCX which is responsible for the carboxylation of specific glutamic acid residues of vitamin K-dependent (VKD) proteins to γ-carboxyglutamic acid^6^. This carboxylation of VKD proteins is an important post-translational modification required for the biological functioning of proteins involved in blood coagulation, bone mineralisation, and calcium homeostasis^7^. When functional defects in the vitamin K-cycle enzymes occur, the VKD proteins are only partially carboxylated, or are not carboxylated at all; these changes to VKD protein’s carboxylation severely affect its physiological functioning^8^.

VKOR uses two cysteines, the CXXC redox motif, as the active site for KO reduction^9^. The active site CXXC is conserved in all VKOR homologues in mammals, plants, and bacteria (except in fungi and yeast)^10^. Both cysteines in the CXXC active site motif are essential for the reduction of KO; mutating either one of these two cysteines reduces activity in all VKOR homologues^9,11,12^. Two additional cysteines, the loop cysteines, are present in the loop region between the first and second transmembrane domains (TMDs); these loop cysteines are also conserved in the VKOR homologues^10^. An active site regeneration mechanism has been proposed for the bacterial homologue based on structure-function studies. In the proposed mechanism, the loop cysteines transfer electrons between the CXXC motif and a thioredoxin-like domain^13–16^. A similar active site regeneration mechanism has been proposed for mammalian VKOR^17,18^. However, mutating both of the loop cysteines in VKOR does not affect its enzymatic activity suggesting that these two loop cysteines are not required for active-site regeneration^11,19,20^.

In addition to the disputed role of the loop cysteines, there are two different proposed models of VKOR’s membrane topology; one model of VKOR as a 3-TM protein^20,21^ and one as a 4-TM protein^18^. The 4-TM model is compatible with the well-known bacterial VKOR homologue; this model proposes that both the amino terminus (N-terminus) and the carboxyl terminus (C-terminus) are located in the cytosol and that both the loop and the active site cysteines face the ER lumen. The 3-TM model proposes that the N-terminus and the active site face the ER lumen, and that the loop cysteines and the C-terminus are located in the cytosol. One of the major differences between the 3- and 4-TM models of VKOR is the orientation of the first TMD. In the 4-TM model, the first TMD has the orientation of N_in_/C_out_ which contradicts the “positive inside rule”^22^, a major factor that controls the orientation of a given TMD. In fact, one can alter the topology of a number of membrane proteins by changing their charged residue distributions flanking the TMD^23,24^. Previous results from our lab support the 3-TM topology model of VKOR and demonstrate that the loop cysteines are unnecessary for active site regeneration^20,21^. Furthermore, computational simulations suggest that the 3-TM model of human VKOR is more stable than the 4-TM model of human VKOR^25^.

Searches for genes homologous to VKOR identified a paralogous protein, VKORL, which shares nearly 50% protein sequence identity with VKOR and can reduce KO as efficiently as VKOR to support vitamin K-dependent carboxylation *in vitro* ^26–28^. VKORL is reported to have a 30-fold higher resistance to VKAs than VKOR and to protect the extrahepatic tissues against the action of VKAs^27^. Therefore, it has been proposed that VKORL rescues VKOR activity in extrahepatic tissues during anticoagulation therapy. Although VKORL supports the reduction of KO *in vitro*, it does not appear to rescue VKOR activity, as it failed to rescue the VKOR-specific production of functional VKD clotting factors in VKOR knockout mice and VKOR knockout HEK293 cells^19,29^. Hammed *et al*., who measured the mRNA levels of VKOR and VKORL in various tissues of wild-type mice and VKOR knockout mice using qRT-PCR, report that the expression level of VKORL is unchanged in all tissues tested, indicating that VKOR and VKORL are regulated independently^27^. Furthermore, VKOR expression was found to be 10-fold higher than that of VKORL in liver tissue where the coagulation factors are produced; greater VKORL expression levels are observed in extrahepatic tissues where it could function to rescue VKA therapy.

VKOR has been extensively investigated, but structure-function studies of VKORL are inadequate. In this study, we established the structure-function differences between VKOR and VKORL using a VKOR and VKORL double gene knockout (DGKO) cell-based assay^19^. We used site-directed mutagenesis to introduce mutations at the critical residues in VKOR and VKORL, and observed the effects of these mutations on enzymatic activity and warfarin resistance. Additionally, differences in the disulfide-linked oligomerization between VKOR and VKORL were explored.

## Results

### VKOR and VKORL have different membrane topologies

**Figure 1:**
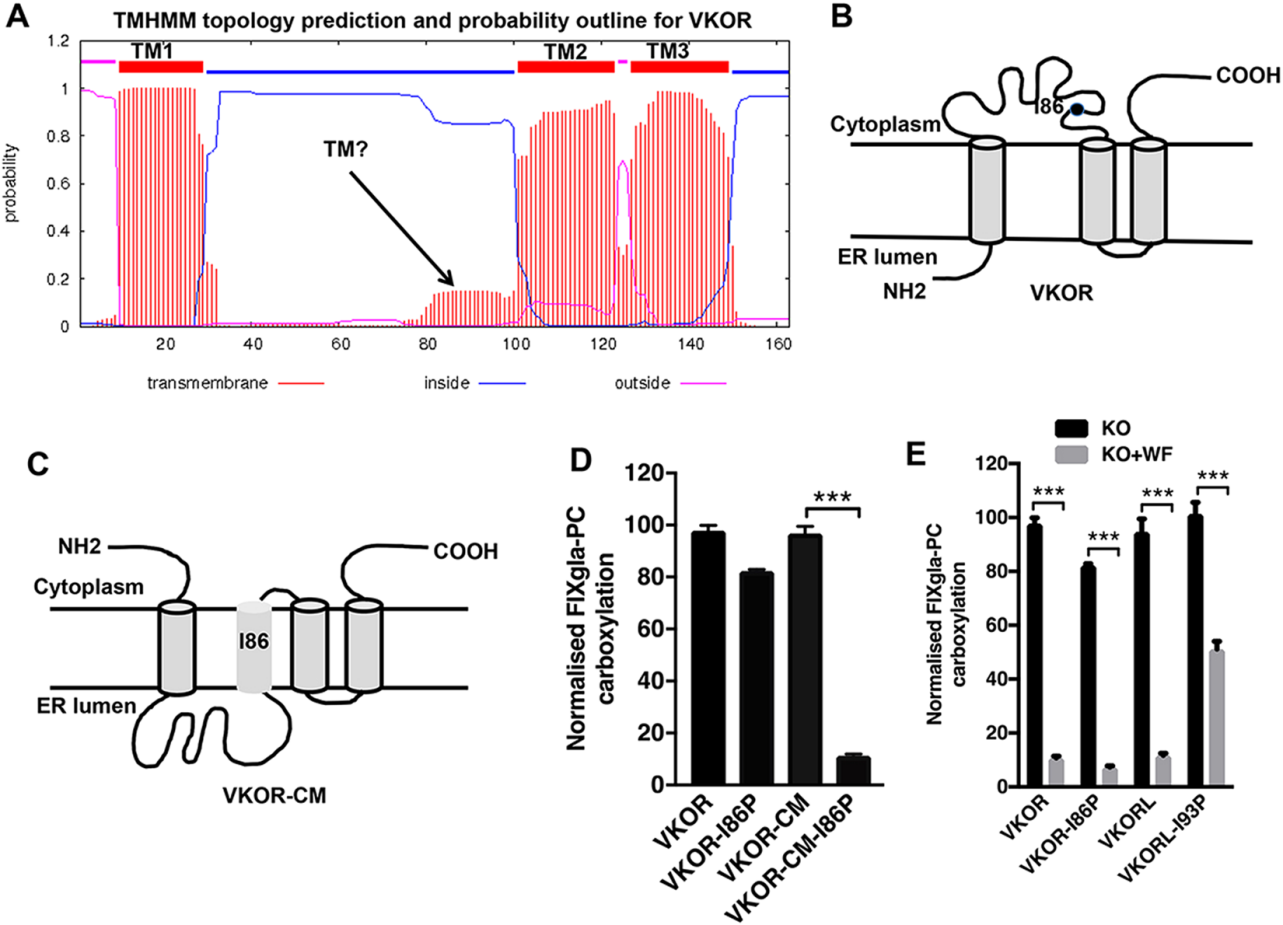
Effect of helix-breaking proline residue on the proposed membrane topologies of VKOR and VKORL. (**A**) TMHMM prediction of VKOR membrane topology. The top line shows the predicted TMDs, and the striped lines indicate the probability of the regions’ functioning as the TMD. (**B**,**C**) Schematic representation of the membrane topology of VKOR and the VKOR charge mutant (VKOR-CM). The location of I86 is indicated. (**D**,**E**) Cell-based activity assay of VKOR, VKORL and their mutants. Wild-type VKOR, VKOR-I86P, VKOR-CM and VKOR-CM-I86P mutants (**D**) were transiently expressed in HEK293-DGKO reporter cells, and the media was supplemented with 2.5 μM KO; 48 hours post-transfection, carboxylated reporter protein FIXgla-PC levels were measured by ELISA. Data is presented as mean ± SD of three independent experiments (n = 3), ***p < 0.001 (unpaired t-test) compared VKOR with VKOR-I86P and VKOR-CM with VKOR-CM-I86P. VKOR, VKOR-I86P, VKORL, and VKORL-I93P mutants (**E**) were transiently expressed in HEK293-DGKO reporter cells, and the cells were grown in media supplemented with 2.5 μM KO in the absence (black bars, KO) or presence (grey bars, KO + WF) of 2 μM warfarin 4 hours post-transfection; 48 hours later carboxylated FIXgla-PC concentration in the cell culture supernatant was measured by ELISA. Data is presented as mean ± SD of three independent experiments (n = 3), ***p < 0.001 (two-way ANOVA), compared to the respective VKOR or VKORL variant with KO and KO + warfarin (WF).

Although it is accepted that VKORL is a 4-TM protein, the membrane topology of VKOR is still under debate^3^. Both 3-TM and 4-TM topological models for VKOR have been proposed. In the 4-TM model, residues 75–97 function as the second TMD (TMD2), while in the 3-TM model, this region is defined as the cytoplasmic loop segment. This segment, if it stood alone, could be integrated into the ER membrane; however, it appears that in the full-length VKOR, this segment’s membrane integration is suppressed by the surrounding sequence^21^. Additionally, the membrane protein topology prediction program TMHMM predicts that the probability of the sequence 75–97 functions as a TMD in the full-length VKOR is small (<0.2) (Fig. 1A). We have previously shown that VKOR can be altered to a functional 4-TM molecule, the VKOR charge mutant (VKOR-CM), by mutating the charged residues flanking VKOR’s TMD1^20^. In the 4-TM VKOR-CM, TMD2 (residues 75–97) has the lowest potential of being a transmembrane helix, and it could, therefore, be easily disturbed for membrane integration. It has been shown that introducing a proline residue into a TM helix can destabilize the membrane protein and affect its function^30^. Therefore, we hypothesized that introducing a proline in the middle of the potential TMD2 (residues 75–97) would disrupt the protein function of only the VKOR charge mutant (VKOR-CM) (Fig. 1C), not of the wild-type VKOR (Fig. 1B). To test this hypothesis, we introduced a I86P mutation into both the wild-type VKOR and the VKOR-CM mutant, and determined the enzymatic activity of each mutant using our cell-based assay. The results show that the I86P mutation has only a minor effect on the activity of VKOR, but it has a dramatic effect on the activity of the VKOR-CM mutant, decreasing its activity to ~10% (Fig. 1D). This result suggests that the potential TMD2 (residues 75–97) does function as an authentic TMD in the VKOR-CM mutant (4-TM model) but not in the wild-type enzyme, supporting the 3-TM model of VKOR.

As a comparison, we tested a similar mutation in VKORL by introducing a proline residue at I93 (the same location as I86, per the sequence alignment). Our results shown that introducing the proline residue did not affect the activity of VKORL (Fig. 1E), perhaps because residues 88–107 function in VKORL as a strong TMD, as predicted by the TMHMM program (probability close to 1.0). However, this mutation significantly increased its warfarin resistance (Fig. 1E). Nevertheless, the warfarin resistance of the I86P VKOR mutant is similar to that of the wild-type enzyme. These results, taken together, suggest that the potential TMD (residue 75–97) segment in VKOR does not function as an authentic TM helix and that VKOR, unlike VKORL, is a 3-TM molecule.

### The P86S mutation in VKORL increased warfarin resistance by 20-fold

**Figure 2:**
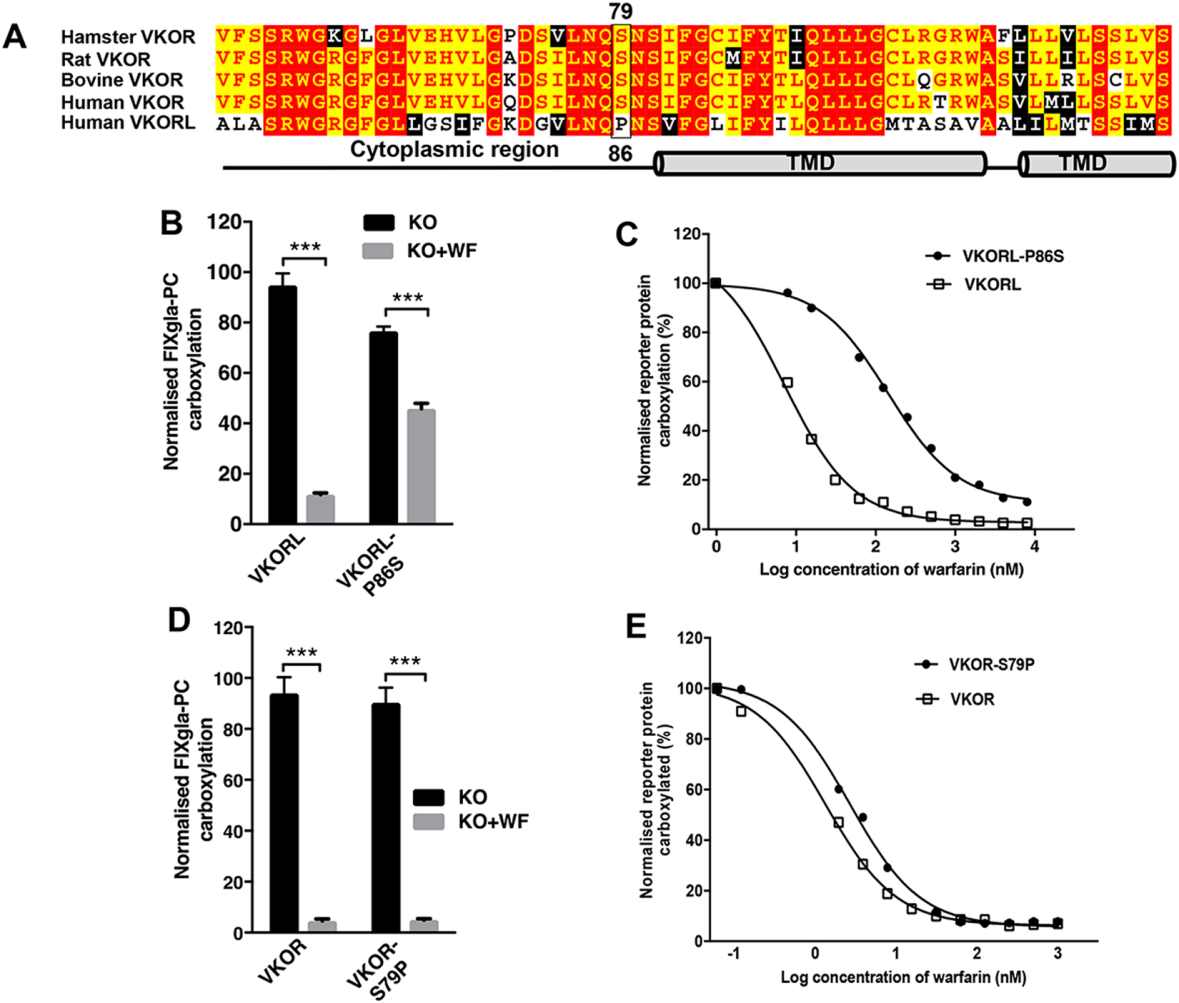
Effect of membrane interface proline residue on the activity and warfarin resistance of VKOR and VKORL. (**A**) Sequence alignment of hamster VKOR, rat VKOR, bovine VKOR, human VKOR and human VKORL. VKOR-S79 and VKORL-P86 residues are represented in boxes. Predicted TM and cytoplasmic regions of VKORL are indicated with graphics. (**B**) Cell-based activity assay of VKORL and its P86S mutant. HEK293-DGKO reporter cells were transiently transfected with VKORL and its P86S mutant and cultured with 2.5 μM KO in the absence (black bars, KO) or presence (grey bars, KO + WF) of 2 μM warfarin 4 hours post-transfection; 48 hours later, the media was collected, and the carboxylation of the FIXgla-PC reporter protein was measured by ELISA to evaluate the enzymatic activity. Data presented as mean ± SD of three independent experiments (n = 3), ***p = 0.001 (two-way ANOVA), compared with VKORL variant with KO and KO + WF. (**C**) Warfarin titration of VKORL and its P86S mutant. VKORL and its P86S mutant were transiently expressed in HEK293-DGKO cells and supplemented with 2.5 μM KO and varying levels of warfarin 4 hours post-transfection. The carboxylated FIXgla-PC concentration in the cell culture media was determined using ELISA after 48 hours. (**D**) Cell-based activity assay of VKOR and its S79P mutant as described in (**B**), HEK293-DGKO reporter cells were transiently expressed with VKOR and the S79P mutant, and media was supplemented with 2.5 μM KO in the absence (black bars, KO) or presence (grey bars, KO + WF) of 2 μM warfarin 4 hours post-transfection; enzymatic activity was measured 48 hours post-transfection. Data presented as mean ± SD of three independent experiments (n = 3), ***p < 0.001 (two-way ANOVA), compared with VKOR variant with KO and KO + WF. (**E**) Warfarin titration of VKOR and its S79P mutant. VKOR and its S79 mutant were transiently expressed in the HEK293-DGKO reporter cells and the cells were cultured with 2.5 μM KO and increasing levels of warfarin; 48 hours later, enzymatic activity was measured as described above.

The sequence alignment between human VKORL and VKOR from different species shows that VKORL has a proline residue at position 86, while in VKOR, this position has a conserved serine (S79) (Fig. 2A). Residue P86 is located at the membrane interface of the N-terminus of TMD2 in VKORL, in a similar location for residue S79 in the 4-TM model of VKOR. While in the 3-TM VKOR model, residue S79 is located in the cytoplasm. Proline residues at the membrane interface of the TM helices often play an important role in correctly orienting essential residues for protein function by facilitating the formation of helix kinks and/or swivel angles^31,32^. We thought that the proline residue of P86 in VKORL might contribute to its function. If this is the case, the conserved residue S79 should have a similar function for the 4-TM model VKOR but not the 3-TM model VKOR. To test this hypothesis, we mutated P86 to serine in VKORL and S79 to proline in VKOR, and then determined the mutants’ activity and warfarin resistance using our cell-based assay. Our results shown that the P86S VKORL mutant had slightly decreased activity, but significantly increased warfarin resistance (~20-fold increase over the wild-type enzyme; Fig. 2B and C). The enzyme activity and warfarin resistance of the S79P VKOR mutant, however, was comparable to that of the wild-type enzyme (Fig. 2D and E). Together, these results support the notion that P86 in VKORL is located in the ER lumenal interface of TMD2 on the same side as the active site; S79 in VKOR is not located at the membrane interface. Thus, these results support the 3-TM model of VKOR and the 4-TM model of VKORL.

### Effect of sequences between the two conserved loop cysteines on the enzymatic activity of VKOR and VKORL

**Figure 3:**
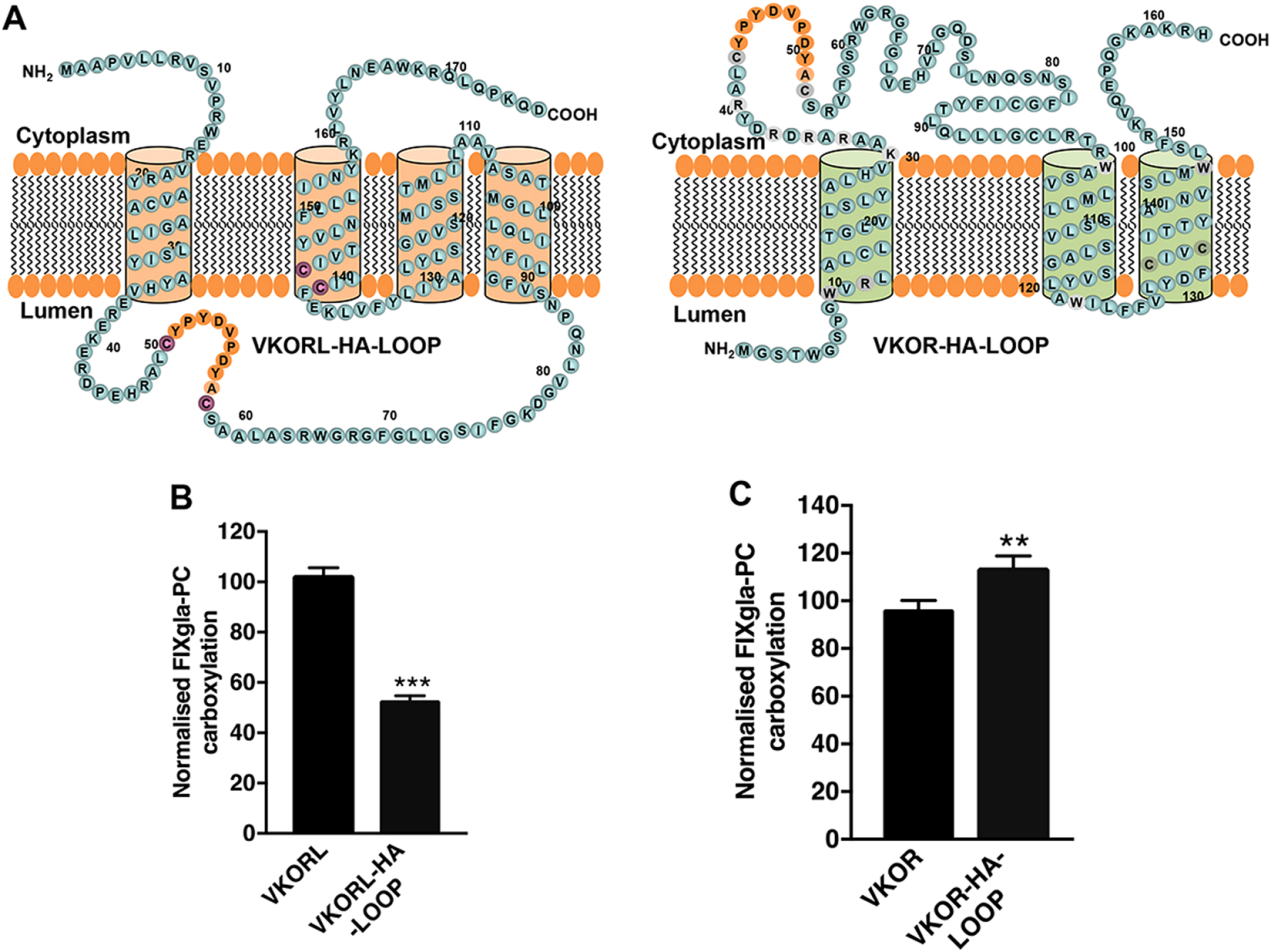
Exchanging the loop region between two conserved cysteines for an HA tag reduced the activity of VKORL. (**A**) Schematic illustration of VKOR and VKORL with their loop regions exchanged for HA tags (YPYDVPDYA). (**B**) Cell-based activity assay of VKORL and the VKORL-HA-LOOP mutant. VKORL and VKORL-HA-LOOP were transiently expressed in HEK293-DGKO reporter cells, and the media was supplemented with 2.5 μM KO. Forty-eight hours post-transfection, protein activity was determined by measuring the carboxylation of the reporter protein FIXgla-PC using ELISA. Data is presented as mean ± SD of three independent experiments (n = 3), ***p < 0.001 (unpaired t-test) compared VKORL with VKORL-HA-LOOP (**C**), VKOR and VKOR-HA-LOOP were expressed in HEK293-DGKO cells, and the media was supplemented with 2.5 μM KO; 48 hours post-transfection, protein activity was measured by ELISA. Data is presented as mean ± SD of three independent experiments (n = 3), **p = 0.002 (unpaired t-test) compared VKOR with VKOR-HA-LOOP.

The different membrane topologies of VKOR and VKORL place the two conserved loop cysteines on different sides the ER membrane (Fig. 3A). We have shown that the conserved loop cysteines of VKOR (C43 and C51) are located in the cytoplasm, on the opposite side of the ER membrane from the active site cysteines; mutating both cysteines of VKOR does not significantly affect the enzyme activity^20,26^. However, the conserved loop cysteines of VKORL (C50 and C58) are located in the ER lumen, on the same side as the active site cysteines. VKORL’s loop cysteines are essential for its activity and are involved in active site regeneration^26^. To further examine the role of the loop region between the two conserved loop cysteines in both proteins, we replaced the loop sequences of VKOR and VKORL with a HA tag sequence (YPYDVPDYA; Fig. 3A) and tested the activity of the resultant mutant proteins using our cell-based assay. We reasoned that if the conserved loop cysteines are involved in transferring electrons to the enzyme’s active site, replacing the sequences between the two cysteines would affect the protein’s enzymatic activity. Our results indicate that the activity of the VKORL-HA-LOOP mutant is reduced to ~50% (Fig. 3B). Conversely, replacing the loop region in the VKOR with the HA tag sequence (VKOR-HA-LOOP) slightly increased the activity of the mutant protein (Fig. 3C). These results suggest that mutating the loop region of VKORL affects its enzyme activity; supporting the idea that the conserved loop cysteines of VKORL are involved in its active site regeneration.

### VKOR, but not VKORL, has a characteristic ER retention signal

**Figure 4:**
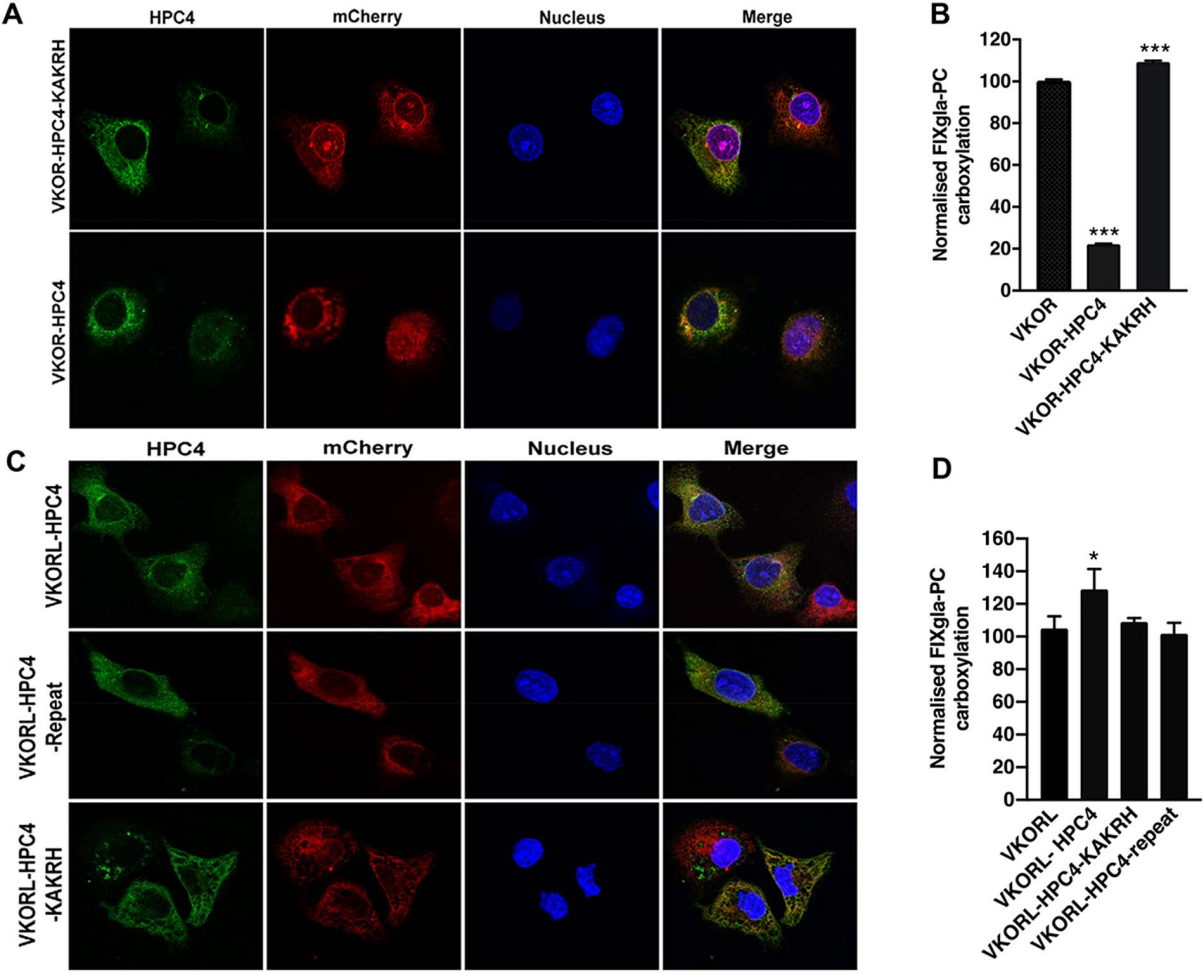
ER retention signal KAKRH is essential for VKOR activity and localisation. (**A**) Immunofluorescence confocal imaging of HPC4 tagged VKOR. VKOR-HPC4 or VKOR-HPC4-KAKRH was co-expressed with the ER marker mCherry-ER-3 in Cos-7 cells. Forty-eight hours posttransfection, transfected cells were fixed with paraformaldehyde, permeabilised with Triton X-100, and immune-stained with mouse anti-HPC4 as the primary antibody and Alexa Fluor 488 conjugated donkey anti-mouse IgG as the secondary antibody. Cell nucleus was stained with Hoechst 33342. Due to the significant low signal of the green channel for VKOR-HPC4 fusion, the Gain of the green channel was increased from 600 (for VKOR-HPC4-KAKRH construct) to 760. (**B**) Effect of the ER retention signal on VKOR activity. VKOR, VKOR-HPC4 and VKOR-HPC4-KAKRH were transiently expressed in HEK293-DGKO cells and cultured with 2.5 μM KO; 48 hours post-transfection, carboxylation of the secreted FIXgla-PC was measured by ELISA. Data is presented as mean ± SD of three independent experiments (n = 3), ***p = 0.001 (unpaired t-test) compared with VKOR. (**C**) Immunofluorescence confocal imaging of HPC4 tagged VKORL. VKORL-HPC4, VKORL-HPC4-Repeat, or VKORL-HPC4-KAKRH was co-expressed with the ER marker mCherry-ER-3 in Cos-7 cells. Forty-eight hours post-transfection, transfected cells were treated as described in (**B**). (**D**) Effect of the ER retention signal on VKORL activity. VKORL, VKORL-HPC4, VKORL-HPC4-KAKRH and VKORL-HPC4 repeat DNA (VKORL’s last eight amino acids RQLQPKQD) were transfected into the HEK293-DGKO cells and cultured in growth medium containing 2.5 μM KO for 48 hours; the carboxylated FIXgla-PC concentration in the cell culture supernatant was then measured by ELISA. Data is presented as mean ± SD of three independent experiments (n = 3), *p = 0.033 (unpaired t-test) compared with VKORL.

The function and structure of VKOR, an ER membrane protein that reduces KO to vitamin K in the vitamin K cycle, is well understood. However, the function of VKORL and its localisation within the cell organelles has not been defined. For example, VKORL has been reported as being localised both within and outside the ER^33,34^. According to a search of the Eukaryotic Linear Motif (ELM) database for VKOR (ELM motif ID: TRG_ER_diLys_1), VKOR has a typical ER retention signal, KAKRH at the C-terminus of the protein. However, there is no characteristic ER retention signal identified for VKORL in the database. To determine the effect of VKOR’s ER retention signal to the protein’s function and subcellular localisation, we engineered an HPC4 tag (EDQVDPRLIDGK) at the C-terminus of VKOR right after the ER retention signal, both with and without an additional KAKRH sequence. We then examined the enzymatic activity and the subcellular localisation of these constructs using our cell-based activity assay and immunofluorescence confocal imaging, respectively. For immunofluorescence confocal imaging, we co-expressed HPC4 tagged VKOR fusions with the ER marker protein mCherry-ER-3 in COS-7 cells. Our results showed that, while the HPC4 tagged protein with an additional KAKRH sequence is clearly localised to the ER membrane. Under the same conditions, the HPC4 tagged protein without the additional ER retention sequence shows a significantly lower fluorescence signal (indicating less intact protein expression) and faint fluorescence signal was observed in the non-ER organelles (Fig. 4A), suggesting the mislocalisation and probable misfolding of the HPC4 tagged protein without the additional ER retention sequence. Results from the cell-based activity assay show that introducing an HPC4 tag directly at the C-terminus of VKOR significantly decreases its activity (to ~20%). However, the HPC4 tagged protein with an additional KAKRH sequence is fully active (Fig. 4B) which is consistent with the immunofluorescence confocal results. These results together suggest that the ER retention signal is essential for the proper targeting and localisation of VKOR.

Similarly, we tested whether adding an HPC4 tag at the C-terminus of VKORL affected its activity and subcellular localisation. We also tested the influence of the ER retention sequence KAKRH as well as the last eight amino acids (RQLQPKQD) of VKORL on the HPC4-tagged VKORL (VKORL-HPC4-KAKRH and VKORL-HPC4-Repeat). Our results showed that VKORL-HPC4 and VKORL-HPC4-Repeat have a similar ER membrane localisation with distinct non-ER distributions (Fig. 4C). The addition of VKOR’s ER retention sequence KAKRH after the HPC4 tag significantly increased the ER localisation of VKORL. Compared with VKOR, significant non-ER distribution was observed for VKORL suggesting a possible non-ER localisation of the native protein. Functional study of these VKORL-HPC4 fusions shows that adding these HPC4 tag sequences does not affect the activity of VKORL (Fig. 4D), which is different from VKOR (Fig. 4B). This suggests that the ER retention signal, which is indispensable to VKOR’s activity, is not essential to the functioning of VKORL. Therefore, VKOR and VKORL could have different locations within the cell.

### VKORL and VKOR form disulfide-linked homo-oligomers

**Figure 5:**
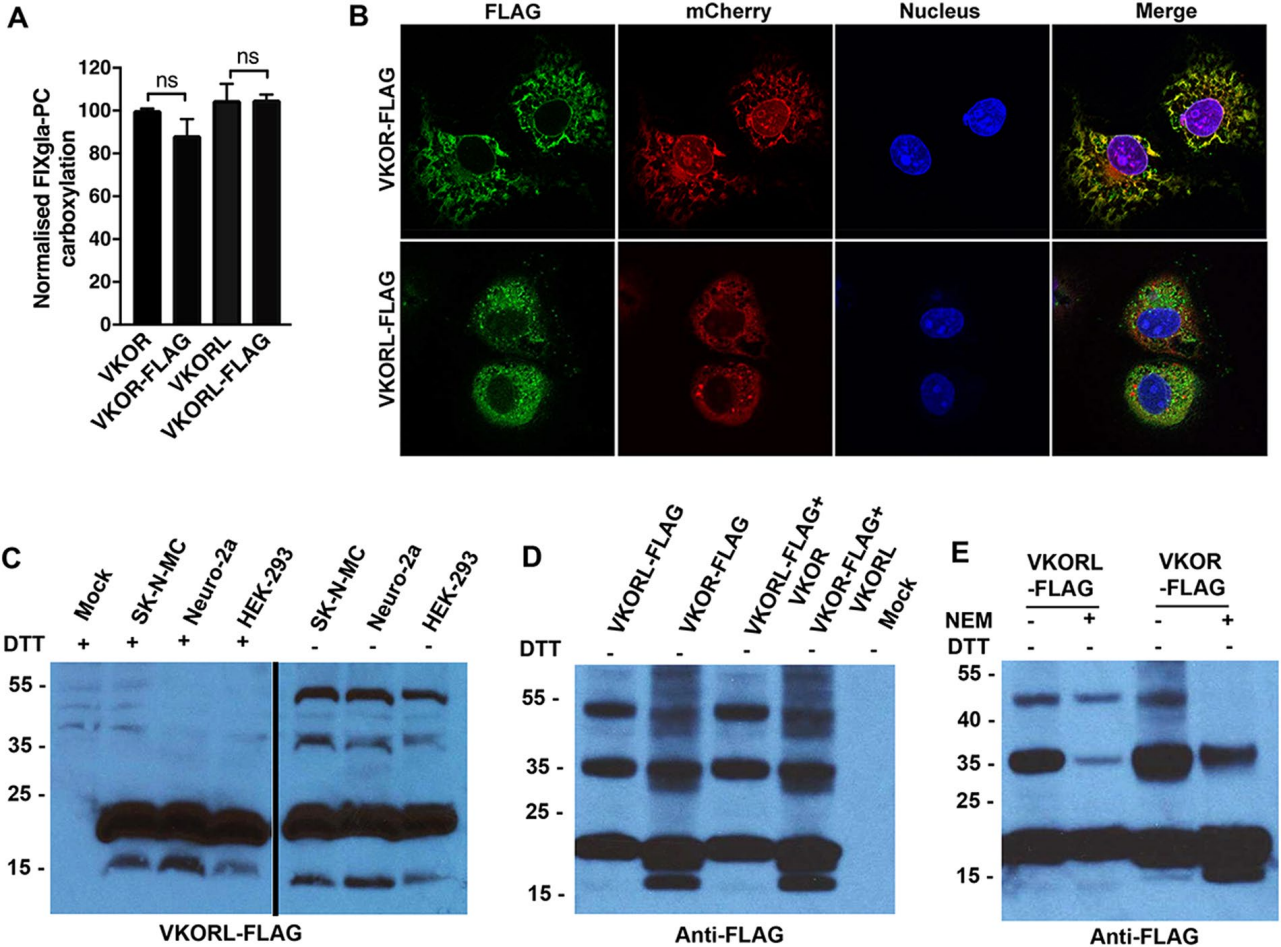
Analysis of disulfide linkage in VKORL and VKOR. (**A**) Cell-based activity assay of the VKOR, VKOR-FLAG, VKORL, and VKORL-FLAG. All constructs were transiently expressed in HEK-293 DGKO cells and the media was supplemented with 2.5 μM KO; 48 hours after transfection, protein activity was determined by quantifying the carboxylation of the reporter protein FIXgla-PC using ELISA. Data is presented as mean ± SD of three independent experiments (n = 3), unpaired t-test was performed to compare VKOR with VKOR-FLAG and VKORL with VKORL-FLAG. The results from the unpaired t-test are not significant. (**B**), Immunofluorescence confocal imaging of FLAG tagged VKOR and VKORL. VKOR-FLAG or VKORL-FLAG was co-expressed with the ER marker, mCherry-ER-3, in Cos-7 cells. Forty-eight hours post-transfection, transfected cells were fixed with paraformaldehyde, permeabilised with Triton X-100, and immune-stained with mouse anti-FLAG and Alexa Fluor 488 conjugated donkey anti-mouse IgG. Cell nucleus was stained with Hoechst 33342. (**C**) Disulfide-linked oligomerization of VKORL in different cell lines. HEK293, SK-N-MC and Neuro-2a cells were transiently transfected with VKORL-FLAG; 48 hours post-transfection, cells were lysed and subjected to Western blot analysis using anti-FLAG antibody under both reducing (+DTT) and non-reducing (-DTT) conditions. (**D**) Western blot analysis of co-expression of VKOR and VKORL. HEK293-DGKO cells were transfected with different constructs as indicated; 48 hours after transfection, cells were lysed under non-reducing conditions, and the input was detected using anti-FLAG antibody. (**E**) Western blot analysis of artificial oligomerization of VKOR and VKORL during the cell lysis. HEK-293 cells were transfected with VKOR-FLAG and VKORL-FLAG and 48 hours post-transfection cell were lysed in the presence of 50 mM NEM and analysed using Western blot under non-reducing (-DTT) conditions. Blots were cropped to improve the clarity and conciseness of the result. The molecular weight marker is indicated to the left of the picture. Mock is the sample from untransfected cells. Full image blots are presented in Supplementary Figure 2A,B and C.

It has been reported that under non-reducing conditions, VKOR exists as a multimeric structure^35^ or forms a hetero-dimer with VKORL^36^. The expression levels of VKOR and VKORL are tissue-specific; VKOR is mainly expressed in the liver, and VKORL is mainly detected in brain tissues^27^. To examine the possible oligomerization of VKOR and VKORL, we fused a FLAG tag at the C-terminus of both enzymes to facilitate their determination by western blot. First, we tested the effect of FLAG tag on the activity of VKOR and VKORL using our cell-based activity assay. Results in Fig. 5A show that adding a FLAG tag at the C-terminus of both enzymes does not have significant effect on their enzymatic activity. Next, we determined the subcellular location of the FLAG-tagged VKOR and VKORL. Our results show that VKOR has a clear ER membrane localisation, while VKORL shows distinct non-ER localisation (Fig. 5B), which is consistent with the results of the HPC4 tagged protein (Fig. 4).

Next, we determined the oligomerization of VKOR and VKORL in different cell types. We expressed both proteins in neural cell lines SK-N-MC and Neuro-2a, and in a human kidney cell line, HEK-293. The western blot result under non-reducing conditions (Fig. 5C) indicated that VKORL forms two disulfide-linked oligomers at ~37 kDa and ~52 kDa. There was no difference in oligomerization between the three cell lines tested. We observed a similar disulfide linkage with VKOR in all three-cell lines (Supplementary Fig. 1). Both of these oligomers disappeared under reducing conditions (in the presence of DTT) (Fig. 5C), suggesting that they are disulfide-linked oligomers.

As VKOR has been co-immunoprecipitated with itself and with its paralogous protein VKORL^36^, we asked whether these oligomers might be due to the interaction between VKOR and VKORL. Therefore, we used HEK293-DGKO cells (endogenous genes of both VKOR and VKORL were knocked out)^19^ to express VKOR and VKORL individually and together, then probed the oligomerization of each of the proteins using Western blot analysis under non-reducing conditions (Fig. 5D). The results indicated that co-expressing VKORL with VKOR showed similar patterns to VKOR or VKORL when expressed alone (Fig. 5D). This suggests that the oligomers of VKOR and VKORL are homogeneous. To determine whether the disulfide-linked oligomerization of VKOR and VKORL exists within the cells in native conditions, we blocked the free cysteines while lysed the cells by adding 50 mM N-Ethylmaleimide (NEM) to the lysis buffer and performed Western blot under non-reducing conditions. Our results showed that the disulfide-linked oligomers of VKOR and VKORL were significantly reduced in the NEM treated samples but, were not abolished (Fig. 5E). Additionally, two forms of disulfide-linked oligomers were observed in the NEM blocked VKORL sample with dramatically reduced intensity; while only one oligomer was observed in the NEM blocked VKOR sample with substantial intensity. These results suggest that cysteines in these two enzymes play different roles, which is consistent with previous studies^20,26^.

### The cysteine residues involved in the disulfide-linked oligomerization in VKOR and VKORL are different

**Figure 6:**
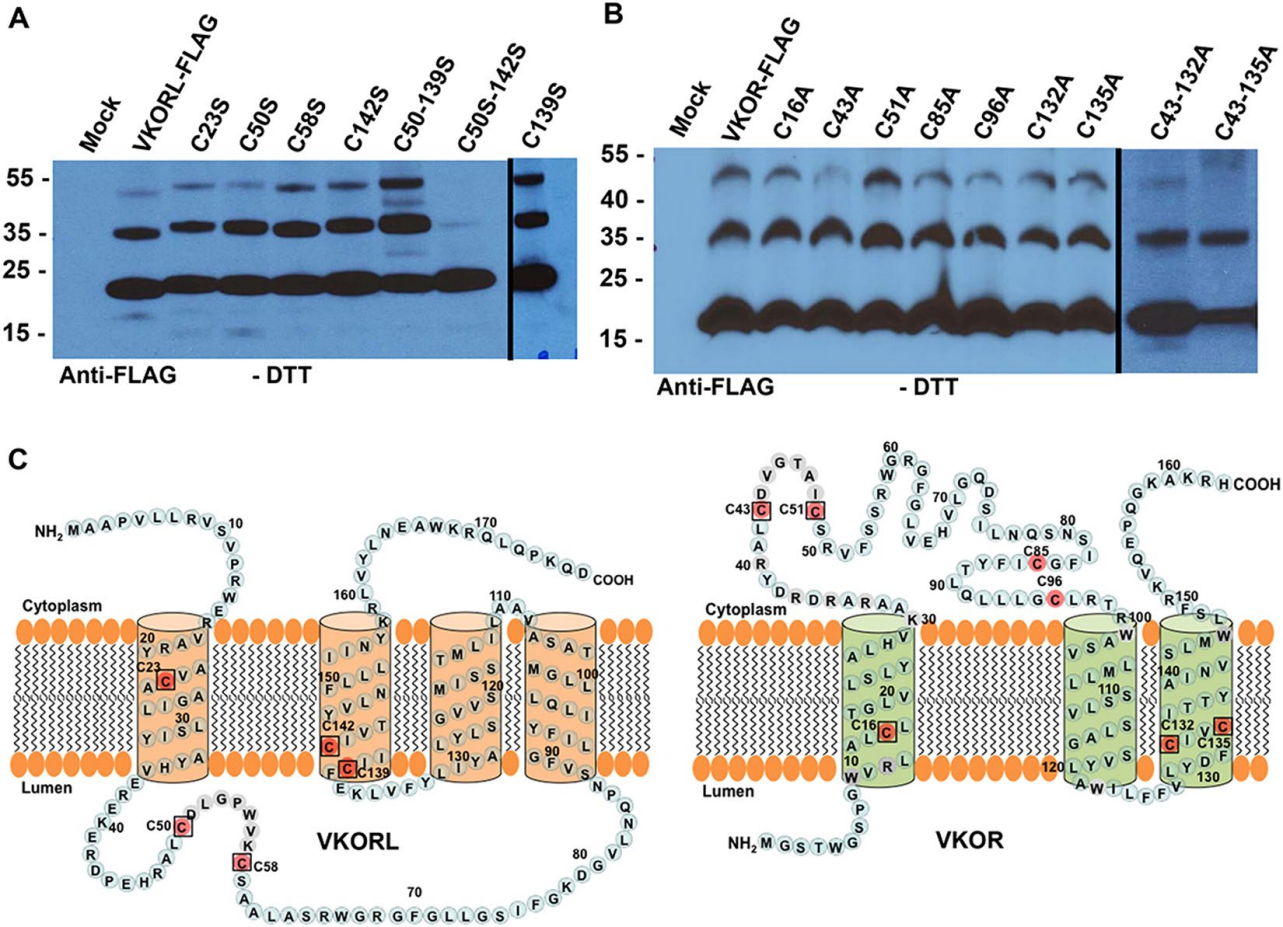
Identification of cysteine residues involved in disulfide linkage in VKOR and VKORL. (**A**) Western blot analysis of oligomerization of VKORL and its cysteine mutants. HEK293-DGKO cells were transfected with VKORL and its cysteine mutants as indicated. Cells were harvested 48 hours post-transfection, and the cell lysates were subjected to Western blot under non-reducing conditions using anti-FLAG antibody. (**B**) Western blot analysis of oligomerization of VKOR and its cysteine mutants. Western blot was performed under non-reducing conditions using the lysate prepared from HEK293-DGKO cells transfected with VKOR and its cysteine mutants as indicated; the input was detected using anti-FLAG antibody. (**C**) Proposed membrane topology cartoon of VKORL and VKOR showing the location of the conserved cysteines. All cysteine residues were coloured red, and the conserved cysteines between VKOR and VKORL are boxed. The figure represents the grouping of different parts from two blots (**A** and **B**), and a black dividing line indicates the spliced image. The molecular weight marker is indicated to the left of the picture. Mock is untransfected cells. Blots were cropped and joined to improve the clarity and conciseness of the result. Full-length blots are presented in Supplementary Figure 3A and B.

To investigate the cysteine residues involved in VKORL’s oligomer formation, we mutated each of the individual cysteines (C23S, C50S, C58S, C139S, and C142S) (Fig. 6A and C) and two cysteine pairs: C50–139S and C50–142S. We chose double cysteine mutation combinations because, as we have previously shown, C58 forms an intramolecular disulfide with C139 during KO reduction^26^. We expressed these mutant proteins in HEK293-DGKO cells and probed oligomerization using western blot analysis. We found that there was no change in VKORL oligomerization after the mutation of any individual cysteine or the C50–139S double cysteine (Fig. 6A). However, mutating the C50 and C142 together to serine abolished the formation of oligomerization, indicating that these two cysteines together are responsible for VKORL’s oligomer formation (Fig. 6A). This result is consistent with the location of C50 and C142 in the oxidative milieu of the ER lumen.

Similarly, we analysed the cysteine residues involved in the disulfide linkages of VKOR by mutating seven individual cysteines (C16A, C43A, C51A, C85A, C96A, C132A, and C135) (Fig. 6B and C) and two cysteine pairs (C43–132A and C43–135A). We expressed these mutant proteins in HEK293-DGKO cells and analysed their oligomerizations using western blot analysis. None of the tested cysteine mutations prevented the formation of oligomers (Fig. 6B). It has been shown that C51 and C132 form an intramolecular disulfide during KO reduction^37^. Therefore, as we did in VKORL, we tested the oligomerization of the VKOR-C51-132A and VKOR-C51-135A mutants. We observed no difference in the disulfide linkage from that of wild-type VKOR (Supplementary Fig. 3). Unlike VKORL, VKOR’s single and double cysteine mutants still form oligomers, indicating that the disulfide linkage patterns by which the VKOR and VKORL form oligomers are different.

## Discussion

VKOR is one of the key enzymes in the vitamin K cycle, which is essential for posttranslational modification of VKD proteins. Although VKORL has a high sequence identity with VKOR, VKORL’s exact physiological function is still unknown. The biochemical characterization of VKORL is proposed to have unrelated physiological functions including acting as an intracellular antioxidant^38^ and rescuing VKOR activity in extrahepatic tissues during anticoagulation therapy^27^. Clarifying the structural and functional differences between VKOR and VKORL could provide insights into their physiological functions.

The structure-function characterization of VKOR has yielded disputed topological structures and mechanisms for active site regeneration. A 4-TM topology model has been proposed for VKOR, based on the crystal structure of its bacterial homologue^18^. However, biochemical and cell-based studies of human VKOR suggest a 3-TM structure^20,21^. The results presented in this study (Fig. 1D and E) are in line with our previous findings indicating that VKOR is a 3-TM protein, unlike VKORL, which appears to be a 4-TM protein. If VKOR were a 4-TM protein, the sequence from residue I75 to L97 would function as the second TMD, and that introducing a TM helix-breaking proline residue between I75 and L97 would likely affect protein function. Results from Fig. 1 show that the I86P mutant has similar activity and warfarin-binding affinity to the wild-type enzyme. However, a similar proline insertion in a VKOR charge mutant (a VKOR molecule with its topology altered to 4-TM^20^) results in a significant decrease in enzymatic activity (to ~10%). In contrast, substituting a proline for VKORL I93 (a similar location to VKOR I86) significantly increased VKORL’s warfarin resistance with only a minor effect on the enzymatic activity; the activity remained stable, which could be because the region where the I93P mutation was introduced was predicted to be a very strong TMD by TMHMM. Introducing a single proline mutation in this location may not be sufficient to break the helix or to have a significant effect on the enzyme activity. Another clear structural difference between the two proteins is the location of certain crucial residues, which has an effect on the protein’s function. VKORL’s proline residue P86 is located near the lumenal ER membrane surface of TMD2, where the reduction of KO occurs; VKOR S79 (the analogous residue to VKORL P86) and the sequences that follow it (which comprise the potential TMD) are located in the cytoplasm of the 3-TM model, on the opposite side of the enzyme’s active site. Mutating VKORL residue P86 increased its warfarin resistance by ~20 fold (Fig. 2C); however, mutating VKOR S79 to proline had no effect on VKOR’s activity or warfarin resistance. Together, these results support our proposition that VKOR and VKORL are structurally different.

VKOR appears to carry a characteristic ER retention signal, KAKRH, which must be at the end of the protein for proper functioning. Covering this ER retention signal by fusing an HPC4 tag sequence at the end of the KAKRH sequence (meaning that KAKRH was no longer identifiable as being in the correct location) dramatically decreased VKOR activity (Fig. 4B). However, the decreased VKOR activity was restored by adding another ER retention sequence, KAKRH, after the HPC4 tag sequence. In contrast, VKORL has no predicted ER retention signal; fusing an HPC4 tag sequence at VKORL’s C-terminal, adding an additional KAKRH sequence or part of the C-terminal sequence of VKORL after the HPC4 tag sequence does not affect VKORL’s activity. These differences suggest a different localisations of these two proteins in the physiological conditions, which agree with the previously reported non-ER localisation of VKORL^33,34^, implying that VKORL may have a different physiological function than VKOR.

Our previous findings indicated that VKORL’s active site is regenerated by transferring electrons from the loop cysteines^26^. This concept is further supported by the results of the current study, which show a 50% reduction in VKORL’s enzymatic activity when the amino acid residues between the loop cysteines are exchanged with the HA tag sequence (Fig. 3B). A similar exchange in VKOR has no effect on the protein’s enzymatic activity, which suggests that the loop region is not essential for VKOR activity; again, this agrees with our previous findings^26^ and indicates that the structure of VKOR differs from that of VKORL.

Our results showed that VKOR could form disulfide-linked oligomers (Fig. 5), which is consistent with the proposed multimeric structure of VKOR^35^. Mutating the cysteine residues to characterise the disulfide linkage revealed that, in VKOR, by mutating each individual cysteine or combinations of double cysteine mutations (C43–132 A, C43–135 A, C51–135 or C51–135), were unable to abolish the disulfide-linked oligomer formation (Fig. 6B). The same test indicated that in VKORL, the double cysteine (C50 and C142) mutation prevents the disulfide-linked oligomerization suggesting that these two cysteines are responsible for oligomer formation (Fig. 6A). These results indicate that both VKOR and VKORL form similar disulfide-linked oligomers, but that the cysteine residues involved in disulfide linkage are different in the two proteins. Although, it is reasonable that the two cysteines responsible for the disulfide-linked oligomerization in VKORL are located in the ER lumen, an oxidizing environment, the logic of the disulfide-linked oligomerization of VKOR is not clear, as none of its cysteines are located in the ER lumen. Whether this disulfide formation has some physiological function in higher species need to be further explored.

Overall, our findings suggest that VKOR and VKORL are structurally and functionally different; suggesting that VKORL may have other physiological functions besides reducing KO to support vitamin K-dependent carboxylation. Further studies are needed to identify the physiological partners/reductants of VKORL to determine its exact cellular function.

## Methods

### Chemicals and Materials

Polyvinylidene difluoride (PVDF) membrane and ECL chemiluminescence reagent were purchased from GE Healthcare Bio-Sciences (Pittsburgh, PA,USA). Mammalian expression vectors phCMV1 and Xfect transfection reagent were bought from Takara Bio USA, Inc. (Mountain View, CA, USA). The Quick-change site-directed mutagenesis kit was from Stratagene (San Diego, California, USA). Q-5 DNA polymerase and T4 DNA ligase were obtained from New England Bio Labs (Ispwich, MA). HEK293, SK-N-MC and Neuro-2a cells were from ATCC (Manassas, VA, USA). Restriction enzymes and pre-stained protein ladders were obtained from Thermo Fisher Scientific (Waltham, MA, USA). Cell culture media, PBS, pBudCE 4.1 mammalian dual expression vector, 4X LDS buffer and NuPAGE™ Novex™ 4–12% Bis-Tris Protein Gels were purchased from Life Technologies (Carlsbad, CA, USA). Fetal bovine serum (FBS) and FLAG monoclonal antibody were from Sigma-Aldrich (St. Louis, MO, USA). Mouse anti-carboxylated FIXgla antibody was from Green Mountain Antibodies (Burlington, VT, USA). Goat anti-mouse IgG and horseradish peroxidase (HRP) conjugated sheep anti-human protein C IgG were from Affinity Biologicals, Inc. (Ancaster, ON, Canada). The 96-well plates for the ELISA, the Hygromycin A antibiotic, and the plastics for the cell culture were from Corning Inc. (New York, NY, USA). The cOmplete^™^ protease inhibitor cocktail tablets were purchased from Roche (Basel, Switzerland).

### Cell culture and transfection

HEK-293, SK-N-MC and Neuro-2a cells were cultured in DMEM F-12 media supplemented with 10% FBS and 1% Penicillin/Streptomycin. HEK293-DGKO reporter cells were cultured in DMEM F12 media with 10% FBS and 0.1 mg/ml Hygromycin A to maintain the stability of the reporter gene, FIXgla-PC. All transfections were performed using Xfect transfection reagent, according to the manufacturer’s protocol.

### Expression vector construction and DNA manipulation

For mammalian cells expression, VKOR or VKORL gene was cloned into the phCMV1 vector with either a FLAG or a HPC4 tag fused at the C-terminus. For C-terminal FLAG tag construction of VKORL, two primers were used: a 5′ primer with a Kozak sequence flanked by the XhoI restriction site, and a 3′ primer with a flexible (GGSG)2 linker, flanked by the FLAG tag (DYKDDDDK) and the KpnI restriction site. For VKOR C-terminal FLAG tag fusion, an additional ER retention signal sequence (KAKRH) was added to the C-terminus of the FLAG tag. Similarly, a HPC4 tag (EDQVDPRLIDGK) was fused to the C-terminus of VKOR or VKORL using a 3′ primer with a flexible (GGSG)2 linker and a HPC4 tag sequence flanked by XhoI restriction site. Additional sequences of the ER retention signal sequence or a short repeat sequence of the C-terminus of VKORL was added to the C-terminus of the HPC4 tag sequence as indicated in the text. To replace the loop sequence between the two conserved cysteines with HA tag (YPYDVPDYA) in the wild-type VKOR and VKORL, we used overlap PCR. For the *in vivo* cell-based activity assay, either VKOR or VKORL gene constructs was sub-cloned into the mammalian dual gene expression vector pBudCE 4.1, between HindIII (5′) and XbaI (3′). No affinity tags were added to the VKOR or VKORL constructs used for the *in vivo* cell-based activity assay. Unless otherwise specified all the affinity tags were added to the C-terminus, and no construct was tagged with two different affinity tags. All the VKOR and VKORL cysteine mutants (Fig. 6A and B) have FLAG tag at the C-terminus.

Site-directed mutagenesis was performed using the Quick-change mutagenesis kit according to the manufacturer’s instructions. All the primers and the DNA sequencing was carried out by Eton Bioscience (Durham, NC, USA).

### Immunofluorescence confocal imaging

Subcellular localisation of HPC4 or FLAG tagged VKOR and VKORL were examined by immunofluorescence confocal imaging. HPC4 or FLAG tagged VKOR or VKORL was transiently co-expressed with the ER marker, mCherry-ER-3 (a gift from Dr. Michael Davidson, Addgene plasmid # 55041), in COS-7 cells on glass coverslips. Forty-eight hours post-transfection, cells were washed three times with PBS and fixed with 4% paraformaldehyde for 15 minutes. Fixed cells were permeabilized with 0.25% Triton X-100 for 10 min. Permeabilized cells were washed three times with PBS and blocked with 2% bovine serum albumin and 5% fetal bovine serum for one hour. Cells were then incubated with mouse anti-HPC4 or anti-FLAG monoclonal antibody in blocking buffer for one hour. After washing three times, cells were incubated with Alexa Fluor 488 conjugated donkey anti-mouse IgG for one hour. Cells were washed again for three times and the cell nuclei were stained with 2 µM Hoechst 33342 (Life Technologies, Carlsbad, CA) in PBS for 20 minutes. Coverslips were mounted onto glass slides with ProLong Gold antifade reagent (Thermo Fisher Scientific, Rockford, IL). Confocal microscopy was performed on a Zeiss LSM710 confocal laser scanning microscope (Carl Zeiss Microimaging, Thornwood, NY). Images were collected using a 40X/1.2NA C-Apochromat objective lens. Visualization of the Hoechst 33342 staining was achieved using a 405 nm diode laser for excitation with the detector set to collect emission at 406–485 nm. Visualization of the Alexa Fluor 488 staining was achieved using a 488 nm argon laser line for excitation with the detector set to collect emission at 505–544 nm. Visualization of mCherry was achieved using a 561 nm diode laser for excitation with the detector set to collect emission at 565–701 nm.

### Detection of FLAG-tagged proteins by immunoblotting

FLAG-tagged VKOR, VKORL or their variants were transiently expressed in one of the following types of cells: HEK-293, HEK-293-DGKO, SK-N-MC or Neuro-2a. Forty-eight hours post-transfection, cells were harvested and lysed in a lysis buffer (150 mM NaCl, 20 mM Tris/HCl (pH 7.4), 1% Triton X-100, 1X Roche cOmplete^™^ protease inhibitor cocktail). Samples were boiled in 1X LDS buffer for 5 min. For some experiments, cells were lysed using the lysis buffer (150 mM NaCl, 20 mM Tris/HCl (pH 7.4), 1% Triton X-100, 1X Roche cOmplete^™^ protease inhibitor cocktail) with 50 mM N-Ethylmaleimide (NEM) to prevent the artificial disulfide linkage. Proteins were separated by sodium dodecyl sulfate–polyacrylamide gel electrophoresis (SDA-PAGE) using NuPAGE™ Novex™ 4–12% Bis-Tris protein gels. After the SDS-PAGE separation, protein bands were transferred onto the PVDF membrane using the wet transfer method for 1 hour at 60 V. The blotted membrane was blocked for 1 hour in 5% non-fat milk in TBST (50 mM Tris-Cl, pH 7.5, 150 mM NaCl, 0.1% Tween) at room temperature, then incubated with anti-FLAG antibody (F3165, Sigma-Aldrich, 1:2000) for 1 hour in the TBST buffer. The membrane was washed (3 × 10 min) with TBST, then incubated with HRP-conjugated goat anti-mouse IgG antibody (GAM-APHRP, Affinity Biologicals, Inc. 1:4000) for an hour. This was followed by another 3 × 10 min wash. The protein signals were then detected using ECL PLUS reagent, and the bands were visualised using Amersham Hyperfilm ECL (28906836, GE Healthcare, USA)

### Cell-based VKOR and VKORL activity assay

The activity of VKOR, VKORL, and their mutants was measured using our cell-based activity assay^39^. This assay was performed in HEK293-DGKO reporter cells, where genes for both VKOR and VKORL were knocked out to eliminate any interference with endogenous KO reductase activity. A chimeric VKD protein, FIXgla-PC (protein C with its Gla domain replaced by that of factor IX), was stably expressed in the DGKO cells as the reporter protein. The functionality of VKOR, VKORL and their variants was measured, similar constructs were transiently transfected in a pBudCE4.1 vector to HEK293-DGKO reporter cells in a 24-well plate using Xfect transfection reagent. Five hours after transfection, the transfection media was exchanged for fresh media with 2.5 μM KO added. The cell culture media was collected 48 hours post-transfection and subjected to sandwich ELISA to measure the levels of carboxylated FIXgla-PC reporter protein, as described previously^39^.

### Warfarin titration assay

For the warfarin resistance study, the VKOR or VKORL genes were transiently expressed in HEK293-DGKO cells. Four hours later, 2.5 µM of KO with increasing concentrations of warfarin was added. The cell culture supernatant was collected 48 hours post-transfection, and carboxylation of the FIXgla-PC protein was measured by ELISA to determine its activity. The half-maximal inhibitory concentrations of (IC 50) values were measured using Graphpad Prism 7.0 software (GraphPad Software Inc., San Diego, CA, USA) to determine the warfarin resistance levels of the VKOR and VKORL mutants.

### Statistical analyses

For each data group, results were expressed as the mean ± SD (standard deviation), and ‘n’ indicates the number of experimental replicates used for the analysis. Statistical analysis was carried out by comparing the VKOR or VKORL mutants with their corresponding control. Differences between the two groups were assessed using the unpaired t-test. A 2way ANOVA was used to determine the differences between more than two groups (Sidak’s multiple comparison test). The statistical significance criteria was set as P < 0.05. GraphPad Prism 7.0 Software was used to perform the statistical analysis (GraphPad Software Inc., San Diego, CA, USA).

### Data Availability

The datasets generated and analysed during the current study are included in this published article

## Electronic supplementary material

Supplementary Figures
