# Supplementary material for: Vitamin K epoxide reductase and its paralogous enzyme have different structures and functions
Source: Sci Rep. 2017 Dec 15;7:17632. doi: 10.1038/s41598-017-18008-3 (PMC5732223; doi:10.1038/s41598-017-18008-3)
Supplement: Supplementary file 1 — Supplementary Figures [file 41598_2017_18008_MOESM1_ESM.pdf]

Supplementary Figures for

Vitamin K epoxide reductase and its paralogous enzyme have different structures and functions

Balaji Chandra Sekhar Sinhadri[1], Da-Yun Jin[1], Darrel W. Stafford[1] and Jian-Ke Tie[1,*]

[1]Department of Biology, University of North Carolina at Chapel Hill, Chapel Hill, North Carolina 27599, United States of America

Corresponding author:

Jian-Ke Tie

Department of Biology

University of North Carolina at Chapel Hill

Chapel Hill, NC 27599-3280

Phone: 919-962-2267

Fax: 919-962-9266

Email: jktie@email.unc.edu

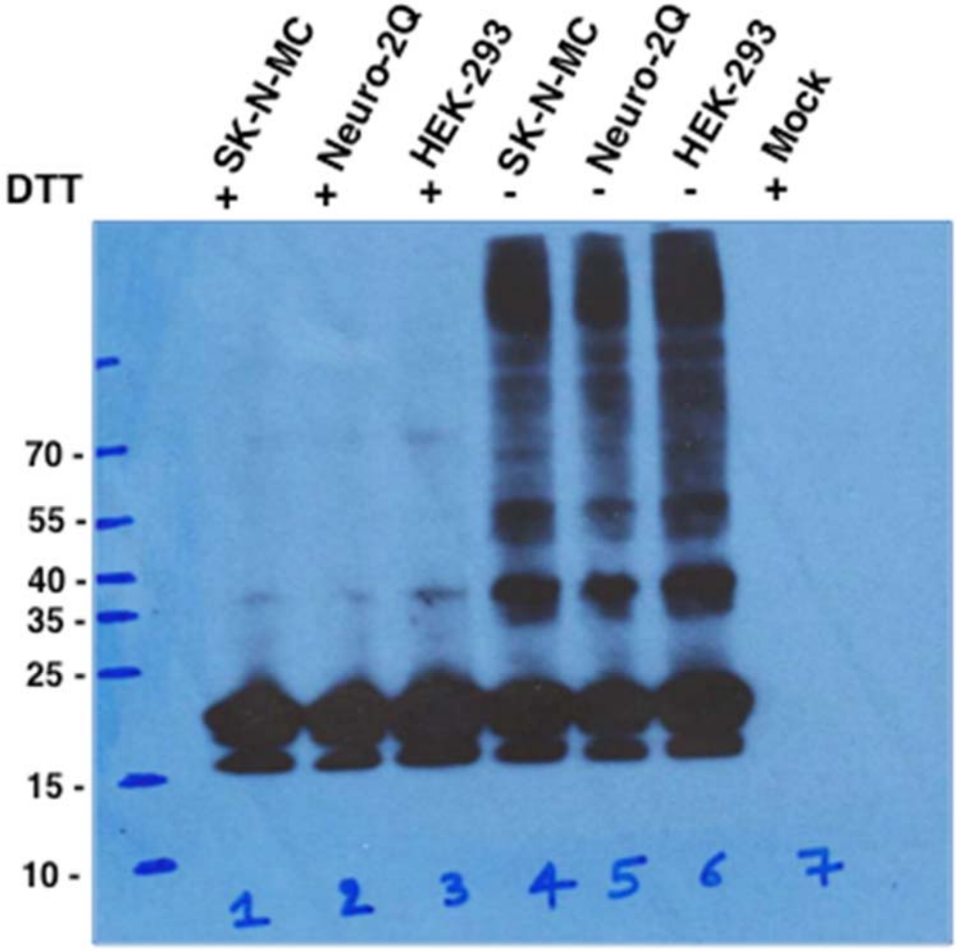

Supplementary Figure 1. Disulfide-linked oligomerization of VKORL in different cell lines. HEK293, SK-N-MC and Neuro 2Q cells were transiently transfected with VKOR-FLAG; 48 hours post-transfection, cells were lysed, and the clarified lysates were subjected to Western blot using anti-FLAG antibody under both reducing (+DTT) and non-reducing (-DTT) conditions. Molecular weight marker is indicated to the left of the picture. Mock is the sample from untransfected cells.

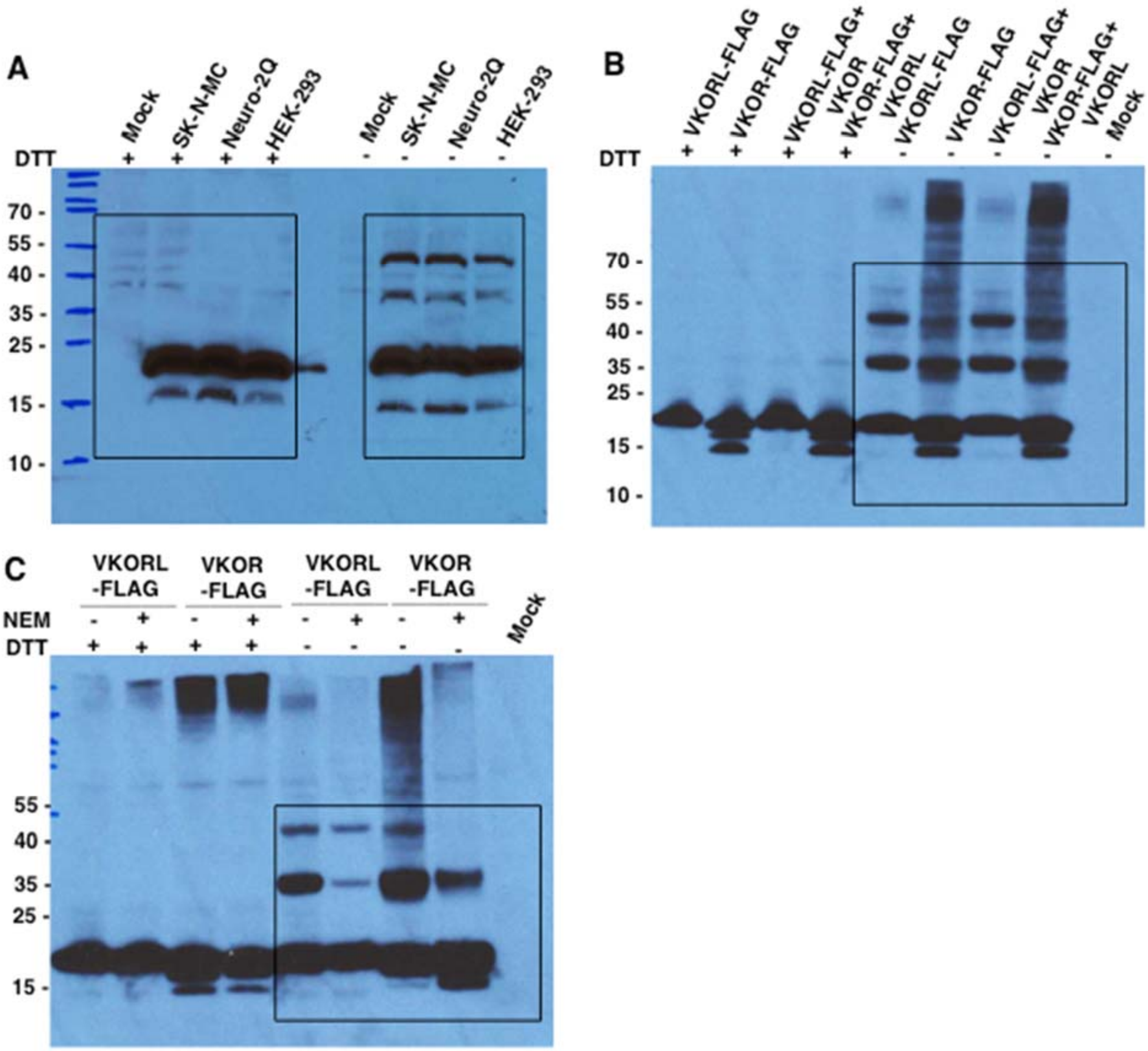

Supplementary Figure 2. Full-length blots represented in Figure 5. Western blot analysis of disulfide linkage in VKORL and VKOR. **A,** Disulfide-linked oligomerization of VKORL in different cell lines. **B,** Western blot analysis of co-expression of VKOR and VKORL. **C,** Western blot analysis of artificial oligomerization of VKOR and VKORL during the cell lysis. Molecular weight markers are indicated on the left side of each blot. Mock is the sample from untransfected cells. Cropped regions of the blots represented in **Figure 5C**, **5D** and **5E** are framed.

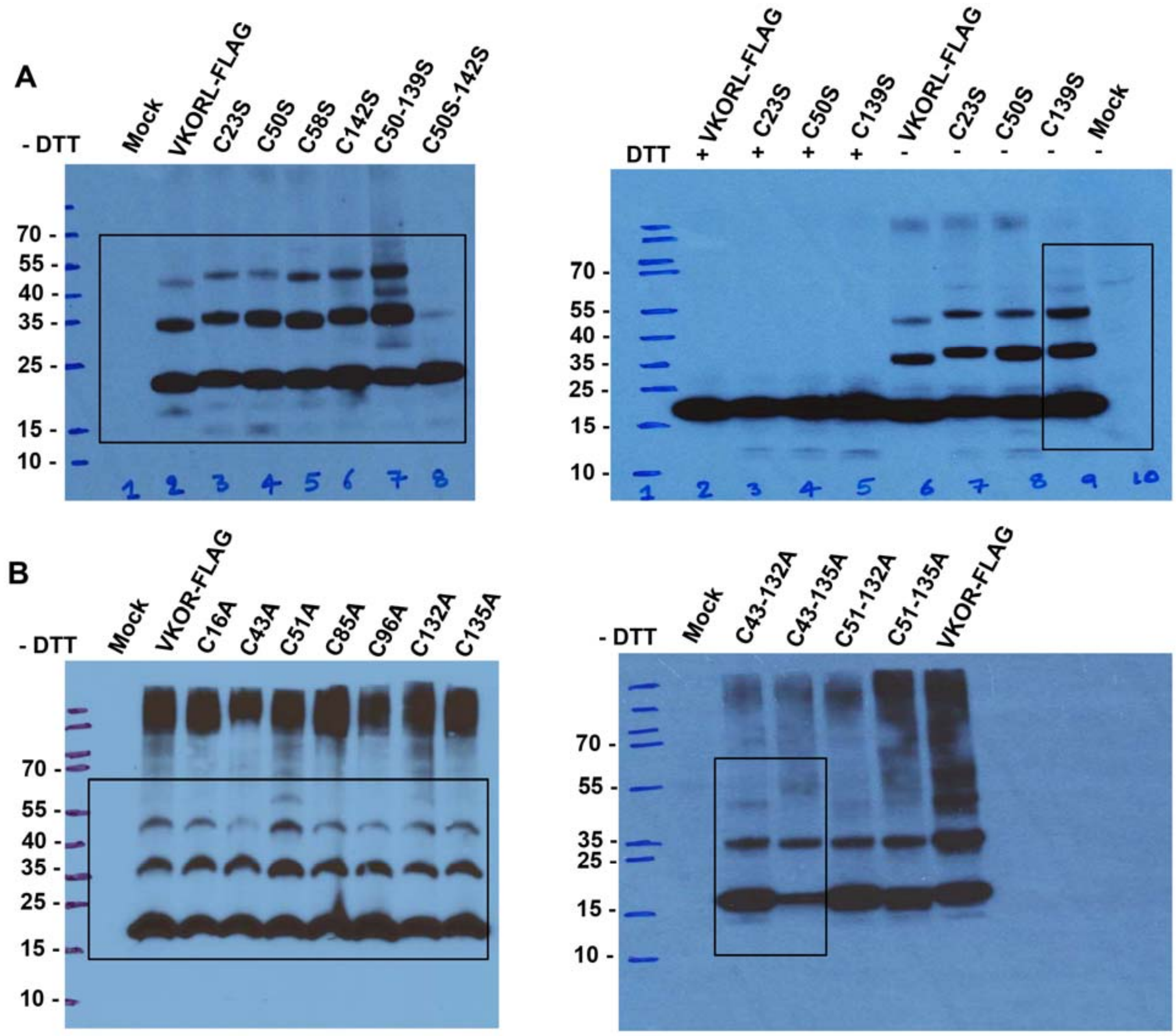

Supplementary Figure 3. Full-length blots represented in Figure 6. Identification of cysteine residues involved in disulfide linkage in VKOR and VKORL. **B,** Western blot analysis of oligomerization of VKORL and its cysteine mutants. **C,** Western blot analysis of oligomerization of VKOR and its cysteine mutants. Molecular weight markers are indicated on the left side of each blot. (-DTT) indicates blots run under non-reducing conditions. Mock is untransfected cells. Cropped regions of the blots represented in Figure **6A** and **6B** are framed.
